# Tables for effective dose assessment from diagnostic radiology (period 1946–1995) in epidemiologic studies

**DOI:** 10.1371/journal.pone.0248987

**Published:** 2021-04-01

**Authors:** Merle Friederike Meiboom, Wolfgang Hoffmann, Kerstin Weitmann, Heiner von Boetticher

**Affiliations:** 1 Center for Radiology and Nuclear Medicine, Gesundheit Nord gGmbH—Klinikverbund Bremen, Bremen, Germany; 2 Institute for Community Medicine, Section Epidemiology of Health Care and Community Health, University Medicine Greifswald, Greifswald, Germany; 3 Division for Medical Radiation Physics, Faculty VI: Medicine and Health Sciences, Carl von Ossietzky University Oldenburg, Oldenburg, Germany; Northwestern University Feinberg School of Medicine, UNITED STATES

## Abstract

Diagnostic radiology is a leading cause of man-made radiation exposure to the population. It is an important factor in many epidemiological studies as variable of interest or as potential confounder. The effective dose as a risk related quantity is the most often stated patient dose. Nevertheless, there exists no comprehensive quantification model for retrospective analysis for this quantity. This paper gives a catalog of effective dose values for common and rare examinations and demonstrates how to modify the dose values to adapt them to different calendar years using a quantification concept already used for retrospective analysis of the red bone marrow dose. It covers the time period of 1946 to 1995 and allows considering technical development and different practical standards over time. For an individual dose assessment, if the dose area product is known, factors are given for most examinations to convert the dose area product into the effective dose. Additionally factors are stated for converting the effective dose into the red bone marrow dose or vice versa.

## Introduction

Diagnostic imaging causes a main part of the radiation exposure of the population in industrial nations [1, 2], although current estimates of the collective effective dose in the population of the United States from medical procedures indicate a decline from 2006 to 2016 of 15 to 20% [3]. Radiation is a risk factor for malignant as well as benign diseases such as leukemia, malignant lymphoma, and solid tumors and also thyroid nodules, eye cataract and impaired brain development [4–8].

Thus retrospective dosimetry is important for individual risk assessment and for analytic epidemiologic studies, as variable of interest or as potential confounder of an association of interest. Therefore it is important to evaluate the lifelong radiation exposure from medical sources of the subjects, where the effective dose represents the most often used quantity.

The effective dose was developed by the ICRP (International Commission on Radiological Protection) as a risk related quantity to estimate human stochastic radiation risk. It represents the radiation risk from a non-uniform radiation dose in terms of a whole body exposure. Mathematically, effective dose is a weighted average of equivalent doses over all organs and tissues of the human body for which specific radiation detriment values can be calculated and tissue weighting factors can be specified [9]. The effective dose is defined for an idealized reference person. Risks for special subgroups of patients or individuals can considerably differ from this average [10].

Nevertheless, the effective dose is often used in epidemiological studies and risk assessments [11]. A more precise way would be considering the particular organ doses, but often it is not possible to get the necessary organ doses.

In order to estimate a lifelong radiation dose it is of great importance, to choose a comprehensive model that adequately considers all relevant factors that might influence patient dose, like calendar year, state of technical improvement, radiological practice and number and spectrum of conventional and advanced examinations.

A detailed quantification concept including all these factors was published by von Boetticher and Hoffmann [12]. It was already applied to the red bone marrow dose [13] but should be adaptive for the effective dose as well, because of its independency of the organ dose concept. Other specific quantification concepts for the effective dose do not exist. In literature only average values for the effective dose are published sometimes according to a defined time period [14].

This paper provides parameters for estimating the effective dose for retrospective studies for the time period 1946–1995. Therefore a catalog of effective doses for common and rare examinations is given. It is demonstrated how these values can be modified by the quantification concept of von Boetticher and Hoffmann [12] to achieve the relevant effective dose for different calendar years. For most examinations factors are given to convert the DAP (dose area product) into the effective dose allowing a more precise estimation of the effective dose for a given DAP. To extend the applicability of the catalog and for comparison purposes additional factors are provided to convert the effective dose into the red bone marrow dose.

## Material and methods

### Model concept

For retrospective dose estimations a model is needed that covers a long time period. Von Boetticher and Hoffmann [12] provided a model of common and rare x-ray examinations for the period 1946–1995. This model accounts for the technical development over this period including the important changes of the equipment as well as the increasing concern for risks of X-ray examinations.

The starting point of this model is the dose for the index period (1976–1985) under optimal conditions including technical equipment, patients characteristics, and radiological practice as it can be expected in an experimental setting (e.g. phantom measurements). These values are then multiplied with two correction factors taken from [12] (Table 1). The first factor (“correction factor for technical advancement”) reflects the technical development over time and depends on the calendar year and the kind of examination. This factor takes into consideration that in the period between 1946 and 1995, continuous technical advances in X-ray devices had a significant impact on patient dose for most types of X-ray examinations. In [12] these factors are derived for X-ray examination, X-ray fluoroscopy, chest X-ray population screening, and computed tomography (CT) (Table 1A–1D). For example, in the case of X-ray films, the patient dose is proportional to the dose required for sufficient exposure of the cassette. Since the 1930’s, technical advancement of the film-screen imaging system allowed for a speed improvement of approximately a factor of 2 in each decade (Table 1A, column 2; factor 1 for the index period 1976–1985) [12]. For X-ray fluoroscopy and chest X-ray screening the most important technical advancement was the introduction of image-amplifier technology. In X-ray fluoroscopy these technique allowed a reduction of the previous screen dose by a factor of 4–5 (Table 1B; column 2), in chest X-ray screening a reduction of the indirect screen procedure by a factor of 20 (Table 1C; column 2). CT became relevant during the decade 1976–1985 and because further technical development was focused on reduction of scan times and higher image quality, the patient dose in CT can be taken as approximately constant (Table 1D; Column 2) [12].

**Table 1:** Matrix of the correction factors for advancement of radiological technology and standard of radiological practice as published in [12, 13]; the most plausible range is indicated.

a. X-ray examination (excluding chest X-ray screening).				
	Standard of radiological practice			
Period	A	B	C	D
until 1945	16	32	64	128
1946–1955	8	16	32	64
1956–1965	4	8	16	32
1966–1975	2	4	8	16
1976–1985	1	2	4	8
since 1986	0.5	1	2	4
b. X-ray fluoroscopy.				
	Standard of radiological practice			
Period	A	B	C	D
until 1965 (chest)	4	8	16	32
until 1965 (abdomen)	5	10	20	40
1966–1975	1	2	4	8
since 1976	1	2	4	8
c. Chest X-ray population screening.				
	Standard of radiological practice			
Period	A	B	C	D
until 1945	16	32	64	128
1946–1955	8	16	32	64
1956–1965	4	8	16	32
1966–1975	2	4	8	16
1976–1985	1	2	4	8
since 1986	0.05	0.1	0.2	0.4
d. Computed tomography.				
	Standard of radiological practice			
Period	A	B	C	D
1976–1985	1	2	4	8
since 1986	1	2	4	8

The second factor (“correction factor for standard of radiological practice”) considers that the real life of radiology practice rarely met the ideal conditions of an experimental setup but usually resulted in systematically higher doses. In [12] correction factors for real life radiology with respect to patient dose are derived considering the relative impact of a multitude of dose-modifying factors from the literature, which usually correspond to higher-than-necessary patient dose. Four standards of radiological practice were defined [12]: A: optimized dose (experimental setup), B: lower realistic dose, C: medium realistic dose, D: higher realistic dose. This second factor also depends on the kind of examination and on calendar year considering the increasing concern for radiation exposure over the time.

To allow the use of this extensive model for the effective doses provided in this paper, the stated values of the effective dose refer to the index period for examinations under optimal conditions. Therefore, the model can be used to estimate the effective dose for the period of the model of von Boetticher and Hoffmann [12] for 1946 to 1995.

In this paper an example is shown as proof of principle for a conventional lumbal spine examination. The values were calculated according to the model of von Boetticher and Hoffmann [12] considering the stated factors in Table 1.

### Effective dose values

Values for the effective dose, DAP and factors for converting the DAP into the effective dose were taken from the literature. Studies considered in the publication of von Boetticher and Hoffmann referred to the time period of interest (Tables 3–7).

If values are stated directly for the index period of 1976–1985 or the next period 1986–1995 these values were taken into account. Generally values of these periods are based on the effective dose concept and the set of weighting factors of ICRP 60 [15]. All data taken from other periods then the index period were corrected in accordance with Table 1. All dose values given in Tables 2–7 are normalized to standard “A” of radiological practice in the index period 1976–1985. Therefore for example data from an X-ray examination in 1986–1995 with standard “A” of radiological practice have to be multiplied by 2 to apply to the index time period (1976–1985) with standard “A”, while the correction factor for the same examination with standard “B” in 1986–1995 is equal to standard “A” in 1976–1985 (Table 1A).

**Table 2:** Mass screening—effective dose (*H*_eff_) and red bone marrow dose (*H*_rbm_) for the index period 1976–1985 and ideal standard of radiological practice (category A according to Table 1).

Sex	Code	DPA	F_eff_	H_eff_	Factor	H_rbm_
		(Gy x cm^2^)	(mSv x [Gy x cm^2^]^-1^)		rbm/eff	(mSv)
Males	1000	**1.556**	0.218	**0.339**	**0.76**	0.256
Females	1000	**1.361**	0.218	**0.297**	**0.92**	0.272

Additionally stated converting factor (*F*_*eff*_) to calculate the effective dose from a given DPA and a factor (*Factor rbm/eff*) to convert the effective dose into the red bone marrow dose and vice versa. (Red bone marrow dose is quoted as stated in the publication of von Boetticher and Hoffmann [12]).

**Table 3:** CT- effective dose (*H*_eff_) and red bone marrow dose (*H*_rbm_) for the index period 1976–1985 and ideal standard of radiological practice (category A according to Table 1).

Type of examination	Code	p	k_CT_	S	CTDI_L_	H_eff_		Factor	Factor	H_rbm_	References
								rbm/eff	rbm/eff		
						Male	Female	Male	Female	Male/Female	
					(mGy)	(mSv)	(mSv)			(mSv)	
Head	2001	1	0.9	1.5	40.45	**0.90**	**1.00**	**4.37**	**4.20**	3.93/ 4.20	Galanski 2001
Neck	2002	1.2	0.9	1.2	27.65	**1.05**	**1.30**	**1.49**	**1.33**	1.56/ 1.735	Galanski 2001
Thorax	2003	1.3	0.8	1.2	23.9	**2.90**	**3.45**	**1.10**	**1.00**	3.18/ 3.455	Galanski 2001
Abdomen	2004	1.3	0.8	1.6	27.4	**5.45**	**7.50**	**0.42**	**0.33**	2.295/ 2.485	Galanski 2001
Lower abdomen, pelvis	2005	1.2	0.8	1.3	30.3	**3.05**	**5.10**	**1.66**	**1.09**	5.06/ 5.535	Galanski 2001
Arms	2006					**0.01**	**0.01**	**2.52**	**2.52**	(0.024/ 0.024)	10 x conventional
Legs	2007					**0.24**	**0.24**	**1.48**	**1.48**	(0.36/ 0.36)	10 x conventional
Hand/ hands	2008					**0.00**	**0.00**	**-**	**-**	0.000 0.000	10 x conventional
Foot/ feet	2009					**0.00**	**0.00**	**-**	**-**	0.000/ 0.000	10 x conventional
Spine	2011	1.1	0.85	1	48.15	**12.40**	**15.00**	**0.62**	**0.56**	7.665/ 8.345	Galanski 2001, Nagel and Galanski 1999
Topogram	2010					**0.10**	**0.10**	**1.00**	**1.00**	0.10/ 0.10	Even et al. 1988
Osteodensi-tometry	2010					**0.13**	**0.13**	**0.30**	**0.30**	0.0375/ 0.0375	Kalender 1995
Liver, kidneys	2010	1.3	0.8	1.9	28	**2.75**	**3.10**	**1.14**	**1.14**	3.145/ 3.525	Galanski 2001
Hip	2010					**1.53**	**2.55**	**1.66**	**1.09**	2.53/ 2.77	1/2 Becken
Shoulder	2010					**0.73**	**0.86**	**1.10**	**1.00**	0.795/ 0.865	1/4 Thorax
Sterno-clavicular joint	2010					**0.73**	**0.86**	**1.10**	**1.00**	0.795/ 0.865	1/4 Thorax
Single vertebra	2010	1.1	0.85	1	48.15	**1.20**	**1.48**	**0.62**	**0.57**	0.745/ 0.835	Galanski 2001

Additionally stated factor (*Factor rbm/eff*) to convert the effective dose into the red bone marrow dose and vice versa. (Red bone marrow dose is quoted as stated in the publication of von Boetticher and Hoffmann [12]).

**Table 4:** Cardiac catheter—effective dose (*H*_eff_) and red bone marrow dose (*H*_rbm_) for the index period 1976–1985 and ideal standard of radiological practice (category A according to Table 1).

Type of examination	Code	DPA_F_	F_eff_	H_eff_	Factor	F_rbm_	H_rbm_	References
		(Gy x cm^2^)	(mSv x [Gy x cm^2^]^-1^)	(mSv)	rbm/eff	(Sv x	(mSv)	
						[Gy x cm^2^]^-1^)		
Diagnostic	4000	**43.2**	0.21	**9.07**	**0.53**	0.1112	4.8	Bernhardt et al.1995, Drexler et al. 1985;
	DIL = 0;8;9							fluoroscopy 50%
Diagnostic plus PTCA	4000	**73**	0.21	**15.33**	**0.53**	0.1112	8.12	Bernhardt et al.1995, Drexler et al. 1985;
	DIL = 0;8;9							fluoroscopy 50%

Additionally stated converting factor (*F*_*eff*_) to calculate the effective dose from a given DPA and a factor (*Factor rbm/eff*) to convert the effective dose into the red bone marrow dose and vice versa. (Red bone marrow dose is quoted as stated in the publication of von Boetticher and Hoffmann [12]).

**Table 5:** Examinations with contrast medium—effective dose (*H*_eff_) and red bone marrow dose (*H*_rbm_) for the index period 1976–1985 and ideal standard of radiological practice (category A according to Table 1).

Type of	Code	DAP	F_eff_	H_eff_	Factor	F_rbm_	H_rbm_	References	different calculation of the extremities for the effective dose compared to the calculation of the red bone marrow dose, Fritz and Koehler 1968
x-ray exam					rbm/eff				
		(Gy x cm^2^)	(mSv x [Gy x cm^2^]^-1^)	(mSv)		(mSv x [Gy x cm^2^]^-1^)	(mSv)		
Gullet (Esophagography)	5001	**8.70**	0.25	**2.18**	**0.66**	0.1646	1.43	Bernhardt et al. 1995;	
								DAP: fluoroscopy 50%	
Stomach (Gastrography)	5002	**24.00**	0.25	**6.00**	**0.12**	0.0305	0.73	Bernhardt et al. 1995;	
								DAP: fluoroscopy 50%	
Small intestine (Selling)	5003	**36.60**	0.3	**10.98**	**0.18**	0.0534	1.95	Bernhardt et al. 1995;	
								DAP: fluoroscopy 50%	
Colon (double contrast)	5004	**41.00**	0.3	**12.30**	**0.18**	0.0534	2.19	Bernhardt et al. 1995;	
								DAP: fluoroscopy 50%	
Pankreas (ERCP)	5005	**22.50**	0.21	**4.73**	**0.15**	0.0305	0.69	Bernhardt et al. 1995;	
								DAP: fluoroscopy 50%	
Gall bladder (Cholecysto-cholangiography)	5006	**22.50**	0.21	**4.73**	**0.15**	0.0305	0.69	Bernhardt et al. 1995;	
								DAP: fluoroscopy 50%	
Kidney (i.v. pyelography)	5007	**20.30**	0.26	**5.28**	**0.12**	0.0305	0.62	Bernhardt et al. 1995	
Arthrography: Shoulder	5008			**0.18**	**0.44**		0.081	H_rbm_: estimated as analogue to (6008)	H_eff_: estimated as 1.5 x (6008)
Arthrography: Knee	5009			**0.007**	**-**		0	H_rbm_: estimated as analogue to (6022)	H_eff_: estimated as 2.5 x (6022)
Arthrographie: Ankle	5010			**0.0001**	**-**		0	H_rbm_: estimated as analogue to (6024)	
Arthrography: Hip	5011			**0.54**	**0.91**		0.493	H_rbm_: 2.5 x (6017)	H_eff_: estimated as 1 x (6017)
								Fritz 1968	
Arthrography: Elbow	5012			**0.0010**	**-**		0	H_rbm_: estimated as (6024)	H_eff_: estimated as 0.5 x (6019)
Arthrography: Hand	5013			**0.000025**	**-**		0	estimated as (6020)	
Arthrography: other joint	5014			**0.007**	**-**		0	H_rbm_: estimated as (6022)	H_eff_: esimated as (5009)
Angiography: Arteries, head	5015	**54.60**	0.0280	**1.53**	**2.69**	0.0754	4.12	Ewen et al. 1982, Vogel 1989	F_eff_ = 6001
	
Angiography: Arteries, chest	5016	**75.00**	0.21	**15.75**	**0.53**	0.1112	8.34	Ewen et al. 1982, Vogel 1989	
								DAP: fluoroscopy 50%	
Angiography: Arteries, abdomen	5017	**65.40**	0.1985	**12.98**	**0.23**	0.0457	2.99	Ewen et al. 1982, Vogel 1989	F_eff_ = 6025A
								DAP: fluoroscopy 50%	
Angiography: Arteries, pelvis	5018	**65.40**	0.29	**18.97**	**0.27**	0.0783	5.12	Ewen et al. 1982, Vogel 1989	F_eff_ = 6018
								DAP: fluoroscopy 50%	
Angiography: Arteries, legs	5019			**1.83**	**2.80**	0.0783	5.12	Ewen et al.1982, Vogel 1989	estimated as 10 x (6021+6023) + 1/12 x (5018)
								DAP: fluoroscopy 50%	
Angiography: Arteries, arms	5020	**0.28**		**0.09**	**1.88**	-	0.169	H_rbm_: 2 x (6008) + whole arm [3 x (6019)]	H_eff_: estimated as 2x(6008)+2*(2*(6019)+(6021))
Phlebography	5021	**5.21**	0.21	**1.09**	**0.37**	0.0783	0.41	Bernhardt et al. 1995;	
								DAP: fluoroscopy 50%	
Hysterosalpingography	5022			**9.48**	**0.27**		2.56	estimated as 0.5 x (5018)	
Myelography	5024	**30.50**	0.345	**10.52**	**0.15**	0.0752	1.59	Bernhardt et al. 1995;	
Pneumoenzepha-lography	5025			**0.76**	**2.69**		2.06	estimated as 0.5 x (5015)	
Bronchography	5026			**15.75**	**0.53**		8.34	as (5016)	
Lymphography	5027	** **		**8.492**	**0.21**		1.81	estimated as 6 x [(6021) + (6023)] + 2 x (6018) + 8 x (6025A) + 4 x (6008); Fritz and Koehler 1968	

Additionally stated converting factor (*F*_*eff*_) to calculate the effective dose from a given DPA and a factor (*Factor rbm/eff*) to convert the effective dose into the red bone marrow dose and vice versa. (Red bone marrow dose is quoted as stated in the publication of von Boetticher and Hoffmann [12]).

**Table 6:** Conventional X-ray examination—effective dose (*H*_eff_) and red bone marrow dose (*H*_rbm_) for the index period 1976–1985 and ideal standard of radiological practice (category A according to Table 1).

Type of	Code	DAP	F_eff_	H_eff_	Factor	H_rbm_	References
x-ray exam					rbm/eff		
		(Gy x cm^2^)	(mSv x [Gy x cm^2^]^-1^)	(mSv)		(mSv)	
Skull	6001	**1.068**	0.0280	**0.030**	**2.69**	0.0805	Bernhardt et al.1995, Drexler et al. 1985
Nose/paranasal sinuses AP	6002	**2.110**	-	**0.055**	**2.94**	0.16	Ewen 1980, Ewen and Lukoschek 1984
eyes	6003	**-**	-	**0.055**	**2.94**	0.16	Estimated as (6002);
Mandible, maxilla	6004	**-**	-	**0.037**	**1.35**	0.05	Ewen and Lukoschek 1984
Mouth (panorama-scan)	6005	**-**	-	**0.008**	**0.38**	0.003	Ewen and Lukoschek 1984
Tooth (single)	6006	**-**	-	**0.007**	**0.57**	0.004	Based on Ewen and Lukoschek 1984
Trachea	6007	**-**	-	**0.342**	**0.44**	0.15	estimated as 0.67 x (6014) + 0.33 x (6015)
Shoulder	6008	**1.464**	0.0840	**0.123**	**0.66**	0.081	Bernhardt et al. 1995
Chest	6009	**1.073**	0.2180	**0.234**	**0.75**	0.175	Bernhardt et al. 1995, Drexler et al. 1985;
							fluoroscopy 27%
Heart, large vessels	6010	**-**	-	**0.670**	**0.76**	0.511	Estimated as (6025E)
Mammography (females)	6011	**-**	-	**0,5**	**-**	0	Jung 2001, Berndhardt et al. 1995
Ribs, osseus thorax (AP/PA)	6012	**1.860**	0.2567	**0.470**	**0.21**	0.097	Stieve and Stender 1990, Drexler et al. 1995
Total spine	6013	**-**	-	**1.200**	**0.88**	1.06	estimated as (6014) + (6015) + (6016)
Neck spine	6014	**1.459**	0.1250	**0.182**	**0.51**	0.0924	Bernhardt et al.1995, Drexler et al. 1985
Thoracic spine	6015	**3.505**	0.1900	**0.666**	**0.40**	0.264	Bernhardt et al.1995, Drexler et al. 1985
Lumbar spine	6016	**9.323**	0.2100	**1.958**	**0.36**	0.701	Bernhardt et al.1995, Drexler et al. 1985
Hip	6017	**3.102**	0.1750	**0.543**	**0.36**	0.197	Bernhardt et al.1995, Drexler et al. 1985
Pelvis (AP)	6018	**3.624**	0.2900	**1.051**	**0.27**	0.284	Bernhardt et al.1995, Drexler et al. 1985
Arm	6019	**1.410**	0.0006	**0.001**	**2.62**	0.0024	Bernhardt et al. 1995
Hand	6020	**0.112**	0.0002	**0.00002**	-	0	Bernhardt et al. 1995
Thigh	6021	**1.200**	0.0056	**0.007**	**5.38**	0.036	Bernhardt et al.1995, Stieve and Stender 1990
Knee	6022	**1.304**	0.0005	**0.001**	-	0	Bernhardt et al. 1995
Lower leg	6023	**0.154**	0.0040	**0.001**	-	0	Bernhardt et al.1995, Stieve and Stender 1990
Ankle	6024	**0.163**	0.0005	**0.0001**	-	0	Bernhardt et al. 1995
Foot	6026	**0.163**	0.0004	**0.0001**	-	0	Bernhardt et al. 1995
Abdomen	6025A	**3.624**	0.1985	**0.719**	**0.15**	0.1105	Bernhardt et al.1995, Drexler et al. 1985
Osteodensitometry	6025B	**-**	-	**0.020**	**0.07**	0.0013	Estimated based on Kalender 1995
„Limbs"	6025C	**-**	-	**0.004**	**4.61**	0.019	estimated as 0.5 x [(6019)+ (6020)+(6021)+ 6023)]
Conventional tomography of skull	6025D	**-**	-	**0.060**	**2.69**	0.161	4 scans; based on Ewen 1980; estimated as 2 x (6001)
Conventional tomography of thorax	6025E	**-**	-	**0.672**	**0.76**	0.511	8 scans; based on Laubenberger 1980; estimated as 4 x (6009 without fluoroscopy)

Additionally stated converting factor (*F*_*eff*_) to calculate the effective dose from a given DPA and a factor (*Factor rbm/eff*) to convert the effective dose into the red bone marrow dose and vice versa. (Red bone marrow dose is quoted as stated in the publication of von Boetticher and Hoffmann [12]).

**Table 7:** Nuclear medicine—effective dose (*H*_eff_) and red bone marrow dose (*H*_rbm_) for the index period 1976–1985 and ideal standard of radiological practice (category A according to Table 1).

Type of exam	Code	Nuclide	Chem. form	Activity	H_eff_	Factor	H_rbm_	References for all values for the effective dose: Reiners and Sonnenschein 1995
						rbm/eff		
				5 y	5 y		5 y	
				15 y	15 y		15 y	
				adult	adult		adult	
				(MBq)	(mGy)		(mGy)	
Skeleton	7001	^99m^Tc	Phosphonate	250	** **	** **	9.5	Reiners and Sonnen-schein 1995
				400			5.2	
				600	**4.8**	**1.20**	5.76	
Lung	7002	^131^I	Microspheres	40	** **	** **	0.6	Reiners and Sonnenschein 1995
				70			0.7	
				100	**1.2**	**0.74**	0.89	
Thyroid	7003				** **	** **		
(until 1976)		^99m^Tc	Iodide	0.8			0.23	Roedler 1986
				1.3			0.23	
(from 1977)				1.9	**21**	**0.01**	0.23	
		^99m^Tc	Pertechnetate	20			0.26	
				35			0.25	
				50	**0.7**	**0.44**	0.31	
Heart	7004							Reiners and Sonnenschein 1995
Ventricle		^99m^Tc	Erythrocytes	300			6	
				500			4.4	
				700	**6**	**0.85**	5.11	
Myocardium		^201^Tl	Chloride	30			20.7	
				55			13.2	
				75	**17.3**	**0.78**	13.5	
Sum					**23.3**	**1.15**	26.7	
							17.6	
							18.61	
Liver/ spleen	7005	^99m^Tc	Large colloides	67	** **	** **	2.55	Roedler 1986
				117			1.76	
				167	**2.2**	**0.84**	1.84	
Kidney (clearance)	7006	^131^J	Hippuran	10	** **	** **	0.06	Reiners and Sonnenschein 1995
				20			0.06	
				25	**0.4**	**0.15**	0.06	
Brain	7007	^99m^Tc	DTPA	185	** **	** **	1.05	Roedler 1986
				324			0.97	
				463	**6.5**	**0.18**	1.16	
Liver/ allbladder	7008	^99m^Tc	HIDA	60	** **	** **	0.78	Reiners and Sonnenschein 1995
				100			0.8	
				150	**3.6**	**0.29**	1.05	
Schilling test	7009	^57^Co	Vit. B_12_	0.008	** **	** **	0.06	Roedler 1986
				0.013			0.05	
				0.02	**0.03**	**1.88**	0.06	
Tumor/inflammation	7010	^67^Ga	Citrate	80			59.2	Reiners and Sonnenschein 1995
				140			35	
				200	**24**	**1.58**	38	

Additionally stated factor (*Factor rbm/eff*) to convert the effective dose into the red bone marrow dose and vice versa. (Red bone marrow dose is quoted as stated in the publication of von Boetticher and Hoffmann [12]).

#### Conventional X-ray examinations: Ideal patient dose for the period 1976–1985

The effective dose (H_eff_) values are mostly derived from Bernhardt et al. [16]. In this publication the effective dose refers to average values of the DAP obtained under typical radiological examinations based on measurements in several German hospitals and private radiological practices in 1992/1993. The DAP are quoted there as well as the used conversion factor (F_eff_) taken from NRPB-R262 [17] which refers to ICRP 60 too. The mathematical formula concerning these values is:

$$H_{eff}=DAP\;x\;F_{eff}$$

*Dental*. The effective dose for dental X-ray examinations including nose, paranasal sinus and facial bones is taken from Ewen and Lukoschek [18] who estimated a somatic dose index for these examinations. This index does not include all organs, included are the lung, the female breast, the thyroid gland, and red bone marrow. Because these organs are the most radiosensitive organs in the irradiation areas of these examinations this can be considered as sufficiently accurate. Average values over both sexes are taken from the sex-specific data provided by Ewen and Lukoschek [18].

*Ribs*. The DAP is taken from Stiever and Stender (time period 1986–1995) [19] as an average value of the ribs 1–7 and 8–12, which is multiplied by 2 to consider the index time period from 1976–1985 (Table 1a). The conversion factor is calculated using the conversion factor for the thorax from Bernhardt et al. [16] multiplied with a correction factor. This correction factor is the proportion of the calculated conversion factors for thorax and ribs taken from Drexler et al. [20] (conversion factor = effective dose / DAP: DAP = field entrance area x surface dose/backscattering factor, the effective dose is calculated as a sum of the organ dose [20] and the conversion dose of the different organs [21]).

The values for shoulder and abdomen are calculated in the same way.

*Extremities*. For the extremities, this paper provided a different way of estimation than this of von Boetticher and Hoffmann [12] because in the basic publication of Bernhardt et al. there are data for the DAP but not instructive data for the effective dose. In this case for the calculation of the effective dose not only the red bone marrow is relevant, which is mainly proximal of the extremities, but also skin, muscle, and bone. The effective dose is calculated from the organ dose (H_org_) and conversions factors (F_con_):

$$H_{eff}=H_{org}\;x\;F_{con}$$

The organ dose (H_org_) is calculated from the skin dose (H_skin_) and the absorption factor (F_abs_) [15]. The skin dose (H_skin_) equals the entrance dose (H_ent_) corrected by the backscattered factor (F_back_ = 1.3) [22].

$$H_{org}=H_{skin}\;x\;F_{abs}$$

$$H_{skin}=H_{ent}\;x\;F_{back}$$

The conversion factor is calculated by the weighting factor according ICRP 60 [15] and the sum of the percentage of the relevant organs (skin, muscle, bone surface and red bone marrow) [ICRP 110 Tab A.1, 23].

The values for females and males are averaged.

#### Contrast medium examination: Ideal patient dose for the period 1976–1985

Examinations with contrast medium are considered as a combination of X-ray films and fluoroscopy. On the basis of the paper of von Boetticher and Hoffmann [12], 50% fluoroscopy is assumed for most examinations (with contrast medium) for the effective dose concept.

The DAP of examinations with contrast medium of the extremities is calculated according to the values of the conventional X-ray examinations corrected by factors taken from Fritz and Koehler [24]. Values for the DAP of the arteriography of the pelvis, abdomen and chest were taken from Ewen et al. [25] and Vogel [26], values from Ewen et al. [25] are considered as realistic standard and therefore are halved to receive standard „A”corresponding to the model applied (Table 1).

#### Computed tomography: Ideal patient dose for the period 1976–1985

Most values for the effective dose for CT-scans (computed tomography scans) were taken from Galanski et al. [27]. The values were halved to receive the values of an optimal practical standard (standard A in Table 1D) due to the consideration that the original values are low realistic standard (standard B in Table 1D).

In contrast to the publication of von Boetticher and Hoffmann [12], this study calculated the values for vertebrae differently due to the consideration that CTDI (computed tomography dose index) and pitch-factor and scanner factor should be equal for single vertebrae and total spine. The effective dose for single vertebrae was estimated as an average value of the cervical and lumbal spine for men and women, respectively. For the effective dose of the spine these values were taken into account considering the total length of the spine for men and women given by Galanski et al. [27].

The effective dose for scans of extremities was estimated as ten times higher than conventional examinations of the same body region. Due to the fact that conventional examinations involved two planes these included twenty single x-ray examinations.

The effective dose values for the topogramm were taken from Evans et al. [28].

Values for the osteodensitometry originate from Kalender [29], the highest dose value was divided in halves to get an average effective dose.

#### Mass screening: Ideal patient dose for the period 1976–1985

Mass screening examinations were conducted as preservation to the spread of tuberculosis. According to Even [28], these examinations of the chest were usually performed without fluoroscopy. Therefore the calculation differs from these of the conventional chest examination. The DPA was calculated from the data of Drexler et al. [30] considering the distance of the x-ray tube, the caliber of the chest, the area product and the entrance dose. Conversion factors were taken from Bernhardt et al. [16].

#### Nuclear medicine procedures and Cardiac catheterization with and without intervention: Ideal patient dose for the period 1976–1985

Values for the dose of nuclear medicine procedures were taken from Reiners and Sonnenschein [31], providing only values for adults.

The effective dose as well as the DPA for the cardiac catheterization with and without interventions was taken from Bernhardt et al. [16].

### Comparison with red bone marrow dose

All values of the red bone marrow dose were taken from von Boetticher and Hoffmann [12]. To convert the effective dose into the red bone marrow dose or vice versa, factors are given as ratio of the red bone marrow dose and the effective dose:

Factorrbm/eff=redbonemarrowdose/effectivedose

## Results

In this paper the effective dose for common and rare radiological examinations was estimated for an index period (1976–1985). The given effective doses are conformed to doses expected under optimal practical conditions that could be expected in an experimental setup / phantom measurement (practical standard A). The results are shown in Tables 2–7. Additionally, factors are given for most examinations to calculate the effective dose if the DAP is known. These factors are shown in Tables 2–7 as well.

In order to estimate a realistic effective dose for an examination of a subject in a given time the quantification model of von Boetticher and Hoffmann [12] can be used. In Table 1 the factors for practical standard and technical standard (calendar year) are stated. The relevant factor has to be identified and multiplied with the stated effective dose of the examination in Tables 2–7.

As an example in Table 8 the realistic dose for a conventional examination of the lumbal spine is calculated according to this model. As stated by the model for the calendar years 1946–1975 the practical standard is C (factor 4) and for the calendar years 1976–1995 is B (factor 2). The taken dose from Table 6 is written in bold and the relevant doses for the different time periods are marked grey.

**Table 8:** Application of the quantification concept of von Boetticher and Hoffmann [12] for an example of the lumbal spine.

Lumbal spine				
calendar year	eff. Dose (mSv)			
	**A**	**B**	**C**	**D**
1946–1955	15.68	31.36	62.72	125.44
1956–1965	7.84	15.68	31.36	62.72
1966–1975	3.92	7.84	15.68	31.36
1976–1985	**1.96**	3.92	7.84	15.68
1986–1995	0.98	1.96	3.92	7.84

The reference dose (optimal radiological practice and time period 1976–1985) as stated in Table 6 is written in bold and the relevant doses for the different time periods are marked grey.

In Tables 2–7, also the organ doses for the red bone marrow are shown as published by von Boetticher and Hoffmann [12]. In order to convert these organ doses into the effective dose or vice versa, factors are provided (organ dose / effective dose).

In case of the computed tomography, the effective dose and the organ dose are comparable in the region of the chest (for example factor rbm/eff 1.1 male / 1.0 female for CT thorax). The head region shows a higher dose for the red bone marrow (factor rbm/eff 4.4 male /4.2 female for CT head) according to the high portion of red bone marrow in the skull. A similar correlation exists for the proximal extremities.

In spite of this, the effective dose is higher in the abdomen scans (factor rbm/eff 0.4 male and 0.3 female for CT abdomen) due to the multiple radiosensitive organs as stomach or intestine.

According to the calculation as an average of cervical and lumbal vertebra, the factors of the spine are an average of the factors for the neck and the abdomen.

The region of the pelvis is the only area that differs between men and women. While the factor for the red bone marrow for women is 1.1, the factor rbm/eff for men is higher with a value of 1.7. Here the genital tract, which is located in the pelvis in women but not men, is considered.

For conventional examinations, the factor rbm/eff in most examinations of the head and the proximal extremities is >1 due to the high fraction of the red bone marrow in these regions.

For the other conventional examinations, the effective dose is higher than the red bone marrow dose and thus the factor rbm/eff is < 1.

Similar factors are provided for examinations with contrast medium: a factor rbm/eff > 1 for examinations of the head and the extremities and < 1 for most of the other examinations. Especially examinations of the stomach and the intestine organs have very low factors rbm/eff (for example 0.12 for stomach examinations with contrast medium).

An outlier in examinations with contrast medium is the phlebography.

While the factor rbm/eff is approximately 1 for CT thorax, the factor rmb/eff for other examinations of the chest (mostly of the heart and mediastine) is smaller, thus the factor rbm/eff for cardiac catheter with and without PTCA is 0.5.

## Discussion

### Effective dose

Our dose catalogue over 1946–1995 is based on a reference period from 1976–1985 and we normalized effective dose values to an optimum standard of radiological practice at that time. The concept of effective dose was introduced in 1975 [32] and included as “effective dose equivalent” into Publication 26 by the ICRP in 1977 [33]. Later, in ICRP 60 [15] the name was shortened to "effective dose" and the tissue weighting factors were revised in ICRP 60 [15] and ICRP 103 [21].

In this study dose values are taken from the literature as far as possible and based on the effective dose concept and the set of weighting factors of ICRP 60 [15]. All data taken from other periods were normalized to an optimum standard of radiological practice in the index period 1976–1985. We preferred effective dose values from literature published as close as possible to our reference period, because these reflect the state of the art of this time. Generally in these publications only the effective dose is given but not the underlying organ dose values.

In ICRP 103, published in 2007 [21], the concept of the effective dose and some organ weighting factors were modified. However, we did not adopt these changes reflecting the more recent different technical background in our retrospective dosimetry to maintain consistency and to allow comparability with data of the earlier periods.

So far only limited parts of the parameters used in our model have been revised or updated. In our methodologic concept allows adopting changes in the factors used for radiologic practice, changes in doses from standard radiologic procedures, and revisions of tissue weighting factors, should these be issued in a future more comprehensive revision.

The effective dose was developed by the ICRP as risk related quantity e.g. it can be used to assess the risks and benefits of the examination to choose an optimal procedure.

The ICRP declares that the effective dose is somewhat limited for individual risk measurements [21]. Nevertheless, it is often used to calculate the radiation risk of subjects in prospective as well as retrospective studies [34–37]. A more precise way would be considering the organ doses, but often it is impossible to get the dose values of the respective organs for the particular examinations. The effective dose model can only be an approximation to any dosimetric approach that is based on the organ doses of all organs exposed by a radiographic exam. Similarly, the estimation of the cancer risk associated with a given exam would preferably be based on the specific organ risks for any respective type of cancer of interest.

This paper provides the framework to estimate the effective dose for retrospective studies. If possible, individual characteristics like gender and age of the subjects as well as available DAP and DLP should be favored to achieve a more precise dose and risk estimation.

Factors are provided to convert the DAP values in the effective dose values to achieve more precise values for the effective dose for a given DAP.

For CT examinations factors converting the DLP into the effective dose are provided detailed in literature [38]. Therefore in this paper only the effective dose is given.

To optimize the estimation of the effective dose for retrospective studies a sufficient quantification model is needed. In this paper the quantification concept of von Boetticher and Hoffmann [12] is used because it adequately considers all relevant factors that might influence patient dose like calendar year, state of technical improvement, radiological practice and number and spectrum of conventional and advanced examinations.

All given effective dose values are standardized for the index period (1976–1985, practical standard A) and can be easily modified by the factors stated in this concept (see example Table 8). This covers a time period between 1945 and 1995 which should be sufficient long for the most retrospective studies including the possibility to calculate the lifelong exposure of subjects.

For the period after 1995 published data should be used due to the fact that it reflects the contemporary technical and practical standard [14, 39]. In the following decades since 1995 national diagnostic reference levels (DRLs) in medical imaging were introduced causing a reduction in variation in dose without compromising the clinical purpose of each examination or procedure. Therefore for these periods it is better to evaluate the time period’s literature as most relevant source for a realistic range of values.

For CT examinations it would be very difficult to estimate sufficient precise factors for the time after 1995 because the technical development with respect to the effective dose is complicated. Thus the dose increases for 4- and 8-detector-scanners compared with single-detector-scanners. This is shown by Gosch et al. [40] who compared different scanners developed by Siemens in the years 1990–1997. The development of scanners with more than 8 detectors leads to a decrease of radiation exposure. Due to the fact that the development of different CT scanners covers a relative short time period there exist many models at the same time. Pantos et al. [39] show that the dose of most CT examinations is constant for the time between 1995 and 2009 except a slight increase of dose for abdomen and pelvis scans for the time after 2006. This constant dose values show that it is difficult to consider the technical development in this time without knowing the used scanner typ. Thus if possible the scanner typ should be found out and taken into account. As Pantos et al. [39] mentioned as well, there can be expected a significant different between countries throughout the world, thus for example development countries probably have older scanners and maybe less experience with radiation protection. In the model of von Boetticher and Hoffmann [12] this could be taken into account by going back one or more 10 year frames. For all CT examinations the dose can be calculated more precisely if the scanner type is known. Unfortunately in most cases this information is unknown.

Mettler et al. [14] provided a catalog of effective dose values. He stated values from studies published between 1980 and 2007 covering phantom measurements as well as large international and national surveys and data from single hospital. This approach results in a wide range for the effective dose of the respective examinations due to technical development in the considered time period and the different practical standards. Thus he had to “make an informed judgment as to what would be a current representative value for effective dose per examination” [14].

In this paper the given effective doses are standardized to a certain time period (1976–1985) and particular setting (phantom measurements). Thus bias due to dose alteration by technical development and practical standards can be reduced. Nevertheless, in practice different technical standards existed simultaneous thus idealizing the dose to a special time period may reduce the variation but does not exclude a deviation by an order of magnitude.

Nevertheless, the values stated by Mettler et al. [14] are comparable with the effective dose values given in this paper considering the overlapping time period. For example for the neck spine Mettler et al. [14] found values between 0.07 and 0.3 mSv and the value in this paper is 0.18 mSv for the time range 1980–2007. Considering the time covered by Mettler et al. [14] and the different practical standards (at least A and B) the range in this paper would be 0.09–0.36 (0.09 for phantom measurements between 1986 and 1995 and 0.36 for common practical standard (B) for the time between 1976 and 1985).

A significant problem concerns the mammography. ICRP 20YY explains “when imaging is limited predominately to one anatomic area, such as in mammography of the breast, estimates of organ or tissue dose should be used instead of effective dose” [9]. Nevertheless for a formal comparison we have taken as base the value of the effective dose for mammography according ICRP 60 from Bernhardt [16] as 0.5 mSv. Considering the remarkable change of the weighting factor from 0.05 to 0.12 in ICRP 103 [21] this “formal” effective dose based on ICRP 103 has be corrected by a factor of 2.4 resulting 1.2 mSv.

### Converting effective dose and red bone marrow dose

The organ dose is defined as a dose absorbed by a particular organ or tissue. It does not include the radiosensitivity of the respective organ or tissue.

Comparing the effective dose with the red bone marrow dose there are considerable differences. In regions with larger quantity of red bone marrow the red bone marrow dose is significant higher (e.g. skull). As expected the opposite is true for regions with low red bone marrow dose but other organs with a high radiosensitivity like in the abdomen.

The factors rbm/eff stated in this paper allow calculating the organ dose for the red bone marrow from a given effective dose. Additionally the red bone marrow dose for the listed examination can be transformed into the effective dose. This could enable the radiologist to compare the risk of different procedures involving not only the red bone marrow dose. Additionally the effective dose is often better known and more familiar to most physicians leading to a better evaluation of the radiation risk for the respective examination.

The factors rbm/eff allow comparing the risks suggested by the effective dose with other possible estimations. One example could be the “lifetime attributable risk of cancer mortality” stated in the BEIR VII report [41]. In further studies this way of estimation could allow to consider the age of the subjects as well as the gender. This would be an advantage compared with the effective dose, which does not consider these individual characteristics.

## Conclusion

In spite of its limitations stated for example by the ICRP 103 the effective dose is an often used quantity in retrospective epidemiological studies due to a lack of practicable alternatives. This catalog provides the effective doses for common and rare examinations and gives an instruction of how to modify the dose values to adapt them to different calendar years using the quantification concept of von Boetticher and Hoffmann [12]. This allows considering technical development as well as different standards in radiological practice. Nevertheless, the limitations of the effective dose concept have to be kept in mind and possible alternatives should be considered. In further studies involving the risk of cancers originating in the red bone marrow the provided factors to convert the effective dose and the red bone marrow dose could be helpful to evaluate the limitations of the effective dose concept.

## List of abbreviations

CT: (computed tomography)
CTDI: (computed tomography dose index)
DAP: (dose area product)
DLP: (dose length product)
F_abs_: (absorption factor)
Factor rbm/eff: (Factor red bone marrow dose/effective dose)
F_back_: (backscattered factor)
F_con_: (conversions factors)
F_eff_: (conversion factor)
H_eff_: (effective dose)
H_ent_: (entrance dose)
H_org_: (organ dose)
H_rbm_: (red bone marrow dose)
H_skin_: (skin dose)
ICRP: (International Commission on Radiological Protection)
PTCA: (Percutaneous transluminal coronary angioplasty)
Rbm: (red bone marrow)
